# Checkpoint Inhibitor-Induced Raynaud’s Phenomenon, Hypothyroidism, and Pneumonitis in Head and Neck Cancer: A Case Report

**DOI:** 10.7759/cureus.109159

**Published:** 2026-05-18

**Authors:** Levente Bodoki, Tamas Csonka, Zoltan Szekanecz, Szilvia Szamosi, Nóra Anna Major, Éva Szekanecz

**Affiliations:** 1 Rheumatology, University of Debrecen, Debrecen, HUN; 2 Pathology, University of Debrecen, Debrecen, HUN; 3 Oncology, University of Debrecen, Debrecen, USA; 4 Oncology, University of Debrecen, Debrecen, HUN

**Keywords:** case report, immune-checkpoint inhibitor, immune-related adverse event, nivolumab, raynaud’s phenomenon

## Abstract

The inhibition of the checkpoint receptor-ligand system has become a therapeutic target in the treatment of solid malignancies in immuno-oncology. Increased attention has been given to immune-related adverse events (irAEs) associated with the use of checkpoint inhibitors. Multiple irAEs may occur in a single patient. Here, we report a unique case in which Raynaud’s syndrome was associated with two other autoimmune complications, hypothyroidism and pneumonitis. The patient was a 48-year-old Caucasian man whose complaints started with a sore throat. Ultrasound and CT revealed left cervical lymph node enlargement. Aspiration cytology showed infiltrating human papillomavirus (HPV)-positive squamous cell cancer. As the outcome of surgery was uncertain, neoadjuvant chemotherapy and irradiation were successfully applied. Later, CT revealed tumor relapse. Nivolumab therapy was initiated, and a total of 21 cycles were administered to the patient. Three months after the initiation of nivolumab, new-onset Raynaud’s phenomenon (RP) developed. No systemic connective tissue disease could be identified. RP improved following oral pentoxifylline treatment. Later, hypothyroidism and pneumonitis developed, both of which were easily controlled. Eventually, significant tumor progression occurred despite nivolumab treatment, and Cetuximab was administered; however, the patient died. It has been established that if two or more irAEs develop in the same patient, one of these irAEs is more likely to be autoimmune rheumatic in nature. It is very important to detect these side effects in time; physicians must be familiar with their appropriate treatment because all side effects may be essential to the patient’s survival.

## Introduction

Inhibition of the checkpoint receptor-ligand system has become a therapeutic target in the treatment of solid malignancies in immuno-oncology [1-4]. Two immune-checkpoint receptor pathways have been most dynamically studied in the context of cancer therapy: cytotoxic T-lymphocyte-associated antigen 4 (CTLA4) and programmed cell death protein-1 (PD-1). Both are inhibitory receptors that regulate immune responses at specific levels and through different mechanisms [1,2]. An important representative of second-generation checkpoint inhibitors is nivolumab, a human IgG4-type anti-PD-1 monoclonal antibody (mAb) [1,2]. 

In recent years, increased attention has been given to immune-related adverse events (irAEs) associated with the use of checkpoint inhibitors [1-3,5-7]. These side effects are more common when using CTLA4 inhibitor ipilimumab, but they also occur with PD-1 and PD-L1 inhibitors. irAEs affect the dermatologic, gastrointestinal, hepatic, endocrine, and other organ systems [1-5]. The most common irAEs for both CTLA-4 and PD-1 therapy are the thyroid, dermatologic, and gastrointestinal toxicities [1,2,5]. However, syndromes resembling classical autoimmune diseases, such as arthritis, myositis, and sicca symptoms, might also occur [1,2,5-7].

The management of such irAEs highly depends on the grading of severity. In mild cases (G1), only symptomatic treatment with corticosteroids or nonsteroidal anti-inflammatory drugs (NSAIDs) should be performed, and immune-checkpoint inhibition can continue. In more severe cases (G2-G4), immunosuppressants, even biologics, should be used along with the cessation or even termination (G4) of cancer immunotherapy [1,2,5,7,8].

Raynaud’s phenomenon (RP) can occur in up to 1% of young females as a primary symptom. It can also be associated with systemic sclerosis and other autoimmune diseases. It should be differentiated from acral vascular syndromes in cancer patients. Moreover, there is a knowledge gap in understanding how RP can develop after anti-PD-1 treatment [1,4].

Our case report is unique, as, while hypothyroidism and pneumonitis relatively commonly occur upon immune-checkpoint inhibition, there have been only very few reports on RP. Also, there have been relatively few reports on triple autoimmune side effects in the same patient. Therefore, we report the development of Raynaud’s symptoms together with hypothyroidism and pneumonitis in a patient treated with the anti-PD-1 monoclonal antibody Nivolumab. Three adverse effects involving three different organ systems required different therapeutic approaches.

## Case presentation

The medical history of this human papillomavirus (HPV)-positive, promiscuous male patient, born in 1967, included clavicle fracture, recurrent sinusitis maxillaris, lumbosacral disc herniation, and left carpal tunnel syndrome. His present complaints began in December 2015 with a sore throat on the left. At the end of December 2015, the patient was referred to the Ear, Nose, and Throat Clinic for consultation. Ultrasound (US) and computed tomography (CT) of the neck revealed left cervical lymph node enlargement. The result of aspiration cytology from the left nuchal and parajugular lymph nodes was squamous cell cancer metastasis. Magnetic resonance imaging (MRI) of the skull and neck revealed a 4 cm long necrotizing parapharyngeal infiltration from the tonsillolingual region that involves the pharynx, plus cervical and supraclavicular lymphadenomegaly on the left side (Figure 1).

**Figure 1:**
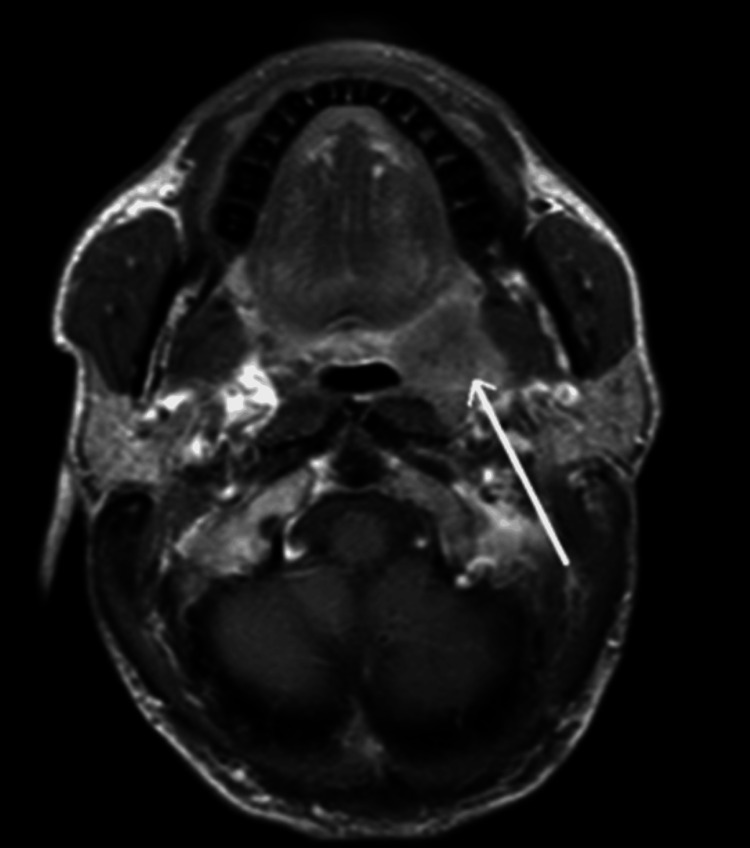
Magnetic resonance imaging (MRI) of the skull, T1-weighted scan. A 4-cm-long necrotizing parapharyngeal infiltration on the left side extending from the tonsillolingual region to the pharynx (white arrow).

At the beginning of January 2016, microlaryngoscopy was performed with histological sampling of this HPV-positive non-keratinizing carcinoma (Figure 2).

**Figure 2:**
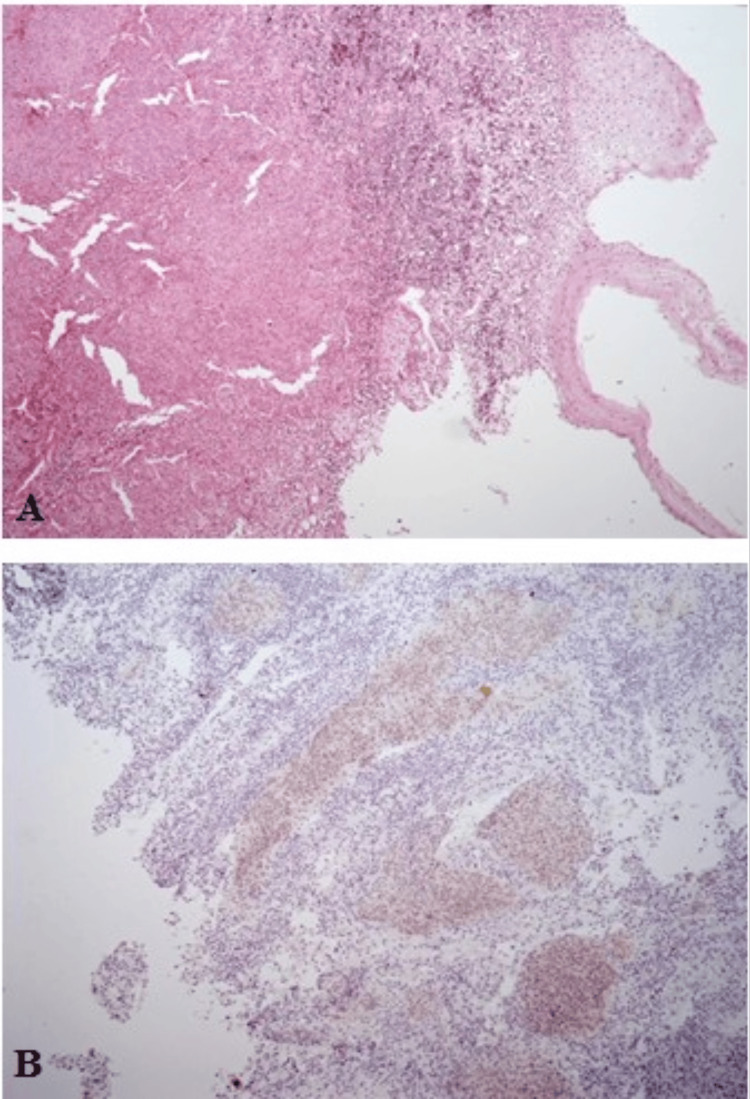
Human papillomavirus (HPV)-positive non-keratinizing squamous cell carcinoma. (A) Hematoxylin and eosin (H&E) staining, 100×; (B) p16 immunohistochemistry, 100×.

According to the decision of the oncoteam, successful surgical removal was questionable because of the location of the tumor, and the patient declined the operation. Therefore, the proposed treatment was neoadjuvant chemotherapy: from May to August 2016, four cycles of docetaxel-containing chemotherapy were administered without any complications. Between September and November 2016, the patient underwent radiotherapy: due to good regression, a total dose of 68 Gy definitive therapy was delivered to the tumor and both sides of the neck. Radiotherapy resulted in several complications: during treatment, percutaneous endoscopic gastrostomy (PEG) implantation was performed to provide nutrition; due to multiple pharyngeal bleeding episodes, the patient required ligation of the left external carotid artery; temporary tracheotomy was also performed. At the end of radiotherapy, the patient had no complaints, quality of life was satisfactory, and repeated restaging tests were negative.

Nine months after radiotherapy was completed, CT confirmed recurrence of the tumor. At the beginning of February 2017, a histological sample was taken again in order to confirm the presence of non-keratinizing squamous cell cancer. The oral and maxillofacial surgeon did not recommend surgery because of the uncertain borders; the radiotherapist stated that no further radiotherapy was possible; and due to the localization, brachytherapy was not feasible. According to the decision of the oncoteam, Nivolumab therapy was recommended on the basis of individual reimbursement. Between October 2017 and August 2018, a total of 21 cycles of nivolumab were administered every two weeks (220 mg IV). Corticosteroids were not administered. Despite the persistence of trismus (lockjaw), left-sided hearing loss, and dysphagia, substantial regression of the lesion was documented. Three months after the initiation of nivolumab, new-onset complaints of RP started (Figure 3). We observed triphasic colour change, while symmetry and digital ulcers were absent. Regarding the differential diagnosis, although other vascular diseases (e.g., paraneoplastic acral vascular syndrome) were also considered, we were convinced that classical RP had developed.

**Figure 3:**
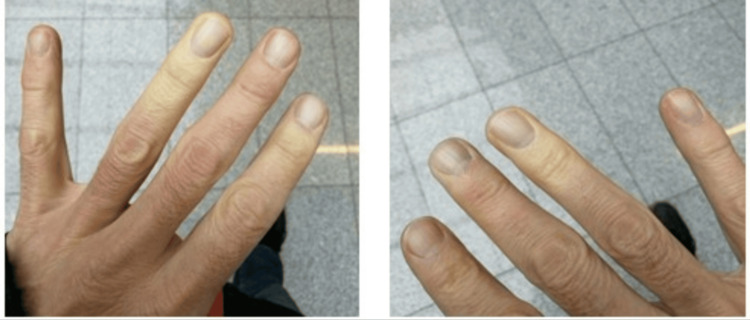
Raynaud’s phenomenon as a side effect of Nivolumab therapy.

At our Rheumatology Outpatient Clinic, blood tests showed no autoantibody positivity, all immunoglobulin levels were within the normal range, and normal capillaries could be seen on nailfold capillaroscopy. The symptoms of RP improved significantly following oral pentoxifylline treatment within a few weeks. One month later, thyroiditis with hypothyroidism was detected during follow-up laboratory tests (sTSH: 47.41 mU/L (normal: 0.4-4.0 mU/L), fT4: 9.5 pmol/L (normal: 10-22 pmol/L), anti-TPO: 645 IU/mL (normal: <16 IU/mL), anti-HTG: 132 IU/mL (normal: <60 IU/mL)). The patient had no clinical symptoms of endocrine disease; with permanent hormone substitution, his hormone levels returned to the normal range. At the end of Nivolumab therapy, new inflammatory abnormalities were detected on chest X-ray and chest CT scans at the bases of the lungs. According to the pulmonologist, the findings were consistent with pneumonitis; however, the patient had no clinical symptoms and therefore did not require treatment. Infectious pneumonia was excluded because CRP and procalcitonin levels were normal, and CT did not show carcinomatous lymphangitis.

At the beginning of September 2018, MRI of the soft tissues of the neck showed significant progression despite Nivolumab treatment (Figure 4).

**Figure 4:**
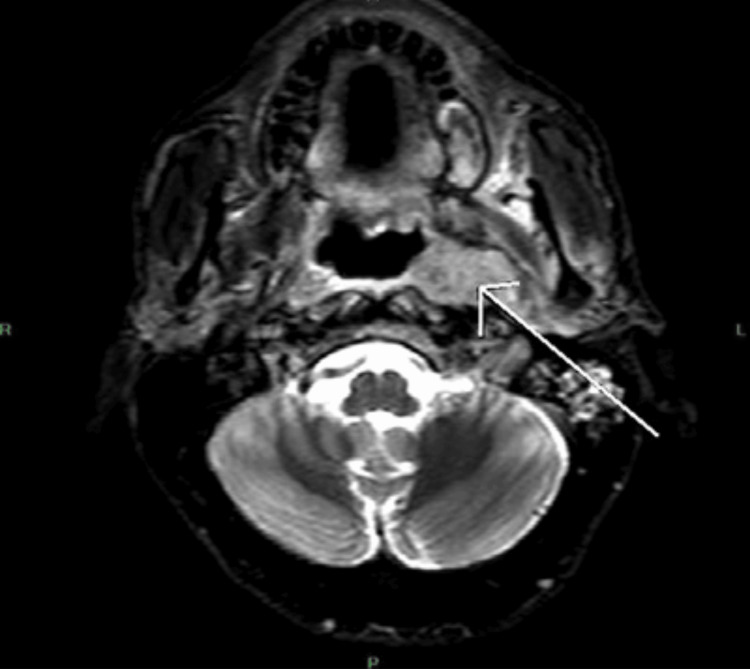
Magnetic resonance imaging (MRI) of the skull, short tau inversion recovery (STIR) sequence. The pharynx, at the level of the mesopharynx, is filled with an approximately 30 × 18 × 33 mm tumor with mixed signal intensity and inhomogeneous contrast enhancement (white arrow).

According to the decision of the oncoteam, a new drug, Cetuximab, was recommended. However, further progression occurred, peritonitis developed, and the patient eventually died in 2019.

## Discussion

Immuno-oncological treatment targets the immune system of the cancer patient rather than the tumor directly. These anti-CTLA-4 and anti-PD-1/PD-L1 antibodies activate antitumor immune responses. When using checkpoint inhibitors, physicians must be aware of new types of irAEs. These irAEs can affect almost every organ system in the body [1-8]. In our patient with head and neck cancer, RP, hypothyroidism, and pneumonitis developed over time during Nivolumab treatment.

The strength of our unique case report is that only very few cases of RP associated with immune checkpoint inhibition have been reported [9,10]. Our case is unique because Raynaud’s syndrome was associated with two other autoimmune complications, hypothyroidism and pneumonitis. It has been established that if two or more irAEs develop in the same patient, one of these irAEs is more likely to be autoimmune rheumatic in nature [1,2]. Possible limitations include the fact that this is a single case report and that other cases with more than one autoimmune adverse event have also been published.

## Conclusions

In conclusion, more than one autoimmune phenomenon, including RP, may occur in a single patient undergoing immune checkpoint inhibition. Early detection of these side effects is very important, and physicians must be familiar with their appropriate treatment, as all of these adverse events may be critical to the patient’s survival.
